# Alterations of bovine nucleus pulposus cells with aging

**DOI:** 10.1111/acel.13873

**Published:** 2023-05-30

**Authors:** Maria Molinos, Morena F. Fiordalisi, Joana Caldeira, Catarina R. Almeida, Mário A. Barbosa, Raquel M. Gonçalves

**Affiliations:** ^1^ i3S – Instituto de Investigação e Inovação em Saúde Universidade do Porto Porto Portugal; ^2^ INEB – Instituto de Engenharia Biomédica Universidade do Porto Porto Portugal; ^3^ ICBAS – Instituto de Ciências Biomédicas Abel Salazar Universidade do Porto Porto Portugal; ^4^ iBiMED – Institute of Biomedicine, Department of Medical Sciences University of Aveiro Aveiro Portugal

**Keywords:** aging, complex analysis, intervertebral disc

## Abstract

Aging is one of the major etiological factors driving intervertebral disc (IVD) degeneration, the main cause of low back pain. The nucleus pulposus (NP) includes a heterogeneous cell population, which is still poorly characterized. Here, we aimed to uncover main alterations in NP cells with aging. For that, bovine coccygeal discs from young (12 months) and old (10–16 years old) animals were dissected and primary NP cells were isolated. Gene expression and proteomics of fresh NP cells were performed. NP cells were labelled with propidium iodide and analysed by flow cytometry for the expression of CD29, CD44, CD45, CD146, GD2, Tie2, CD34 and Stro‐1. Morphological cell features were also dissected by imaging flow cytometry. Elder NP cells (up‐regulated bIL‐6 and bMMP1 gene expression) presented lower percentages of CD29+, CD44+, CD45+ and Tie2+ cells compared with young NP cells (upregulated bIL‐8, bCOL2A1 and bACAN gene expression), while GD2, CD146, Stro‐1 and CD34 expression were maintained with age. NP cellulome showed an upregulation of proteins related to endoplasmic reticulum (ER) and melanosome independently of age, whereas proteins upregulated in elder NP cells were also associated with glycosylation and disulfide bonds. Flow cytometry analysis of NP cells disclosed the existence of 4 subpopulations with distinct auto‐fluorescence and size with different dynamics along aging. Regarding cell morphology, aging increases NP cell area, diameter and vesicles. These results contribute to a better understanding of NP cells aging and highlighting potential anti‐aging targets that can help to mitigate age‐related disc disease.

## Graphical abstract

Main alterations in nucleus pulposus (NP) cells with aging were investigated. Bovine coccygeal discs from young (12 months) and old (10–16 years old) animals were dissected, and NP cells were characterized by gene expression, proteomics, flow cytometry and imaging flow cytometry. Providing a deeper understanding of NP cells aging highlights potential anti‐aging targets that can help to mitigate age‐related intervertebral disc disease.

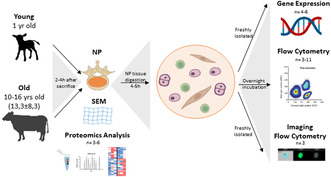

**Abbreviations**
AC: articular cartilage
ACAN: aggrecan
AF: annulus fibrosus
CEP: cartilage endplate
COL2: collagen type 2
DDD: degenerative disc disease
ECM: extracellular matrix
GO: gene ontology
IHC: immunohistochemistry
IL: interleukin
IVD: intervertebral disc
LBP: low back pain
MMP: metalloprotease
NP: nucleus pulposus
SEM: scanning electron microscopy
sGAGs: sulphated glycosaminoglycans
TGF‐b: transforming growth factor‐beta

## 1 INTRODUCTION

According to the last Lancet Global Burden Disease Study, low back pain (LBP) remains one of the leading disorders in years lived with disability, in both developed and developing countries (Vos et al., 2020), constituting a heavy socio‐economic burden in modern society (Maher et al., 2017). Although the aetiology of LBP is still unclear, several reports suggest the existence of a strong positive correlation between degenerative disc disease (DDD) and LBP (Cheung et al., 2009; Teraguchi et al., 2014). Current treatments for DDD neither address the underlying pathogenesis nor seek to restore IVD's function or to slow down disease progression. Notwithstanding, novel regenerative therapies, born from solid cellular and molecular knowledge of the IVD, are actively being sought (Kamali et al., 2021; Richardson et al., 2016; Sakai & Andersson, 2015).

Despite being a cartilaginous tissue, the IVD is heterogeneous and contains different cell types, whose molecular/phenotypic signature remains to be classified (Pattappa et al., 2012). Particularly, for the nucleus pulposus (NP), during the last years, various microarray studies have been conducted in order to better define the phenotypic signature of NP cells, more specifically in relation to the neighbouring annulus fibrosus (AF) or cartilage endplate (CEP) cells, or even to articular cartilage (AC) cells (Minogue et al., 2010; Rodrigues‐Pinto et al., 2013; Rutges et al., 2010). Nevertheless, the markers reported so far are not exclusive to any of those cell types; this wide‐genome analysis alone is not fulfilling the need for specific NP cell markers. In 2015, a consensus paper from the Orthopedic Research Society proposed a panel of markers to define the phenotype of young/healthy NP cells (Risbud et al., 2015). This work has prompted new immunohistochemical studies in larger human cohorts seeking further validation of those markers, and others less explored (Richardson et al., 2017; Thorpe et al., 2016). More recently, taking advantage of single‐cell and transcriptomic analysis, several cell populations were identified in the NP including notochordal cells, mesenchymal‐like cells, NP and AF progenitor‐like cells and myeloid/immune cells (Gan et al., 2021; Panebianco et al., 2021; Tu et al., 2022). Nevertheless, many of these cell subsets have not been isolated and their contribution to the progression of IVD degeneration has yet to be addressed.

The natural aging process represents one of the major factors responsible for disc degeneration and seems to affect this organ in a premature manner, when compared to other tissues of the human body (Boos et al., 2002). Different animal models have been used to investigate the distinct features of disc aging (Daly et al., 2016). Amongst these, the bovine animal model is a widely accepted model to study the IVD due to its cellular and biomolecular resemblance to the human scenario (Miyazaki et al., 2009; Panebianco et al., 2021; Sitte et al., 2014). In particular, bovine and human discs present similarities in terms of: (1) cell density (NP density was found to be low (average 4264 cells/mm^3^) in bovine discs, closed to the value (about 6000 cells/mm^3^) reported for the human (Miyazaki et al., 2009); (2) morphology (similar structure of bovine and human discs); (3) biomechanics (swelling pressure of bovine coccygeal discs similar to that of the discs in a person resting in the prone position); and (4) notochordal cells (bovine discs closely resemble the human discs because adult human and adult bovine NP have almost no notochordal cells and contain chondrocyte‐like NP cells that produce a hyaline cartilage‐like matrix). The notochordal cells are completely replaced by cartilage‐like NP at birth in cattle and after 10 years in humans (Hunter et al., 2003); and (5) biochemical composition (similar rates of in vitro proteoglycans synthesis and matrix synthesis in response to hydrostatic pressure in human and bovine coccygeal discs). Both bovine and human discs are also similar with regard to the types and distribution of aggrecan and collagen (Miyazaki et al., 2009)”.

We have previously shown that NP aging involves changes at structural level, with a decrease in water loss, proteoglycans content, fewer and larger pores in the extracellular matrix (ECM). These changes are accompanied by the loss of collagen type XIV and type XII from foetal stages to adulthood, while with increasing age, there is an enrichment in fibronectin and prolargin (Caldeira et al., 2017). Nevertheless, alterations on the profile of NP cells with age have been poorly monitored and contradictory results have been found between microarray data (gene level) and those collected at the protein levels (immunohistochemistry (IHC) or flow cytometry) (Sakai et al., 2012; Wu et al., 2018). This may be in part due to the difficulties associated with the scarcity and reliability of bovine antibodies in the market or just by the modifications that may occur in IVD cells at post‐transcriptional level. Nevertheless, there is a lack of standardized tools to characterize IVD cells, particularly from bovine origin.

Flow cytometry is a widely used technique to phenotype single cells and characterize subpopulations. Predominantly, it is measured by the fluorescence intensity resulting from fluorescent‐conjugated antibodies directly recognizing target proteins, or ligands specifically binding to molecules within a cell. Flow cytometry data is extremely quantitative and it provides fast, objective and simultaneous multi‐parameter analysis of single cells. Despite this huge potential, flow cytometry has seldom been used to profile IVD cells. In fact, unlike what is routinely done with blood cells, no gating strategy, specific surface markers or Forward‐Scatter/Side‐Scatter (FCS/SSC) (that allows for cell size/granularity) profile has been proposed for IVD cells yet. Previously, we have disclosed three subpopulations of bovine NP cells using flow cytometry, based on cells' distinct size and auto fluorescence, as well as differential expression of the surface markers CD29, CD44 and CD146 (Molinos et al., 2015).

In the present study, we explored high‐throughput analysis of bovine NP cells along aging. For that, initially, the cellulome of bovine NPs from young (approximately 1 year old) and old (more than 10 years old) animals was analysed. Then, bovine NP cells from animals with distinct ages were isolated and characterized by gene expression for inflammatory markers, proteolytic enzymes and ECM proteins, to confirm expected differences between NP cells with age. Then bNP cells were characterized under flow in terms of their morphology and expression of surface markers (that include mesenchymal‐related (CD29, CD44, CD146 and Stro‐1), hematopoietic (CD45 and CD34) and NP‐progenitor (GD2 and Tie2). This study thus contributes to defining the signature of functional bovine NP cell subpopulations and characterizes their alterations with aging.

## 2 MATERIALS AND METHODS

### 2.1 bNP macroscopic characterization and scanning electron microscopy (SEM)

bNP macroscopic and SEM imaging were performed as previously described (Caldeira et al., 2017). Briefly, qualitative macroscopic evaluation of the bNPs from different ages was performed by a Olympus SZX16 stereomicroscope coupled with a DP71 camera (Olympus) at 10× magnification. SEM imaging from bNPs was performed after sample fixing with glutaraldehyde (Agar Scientific) 2.5% (v/v) in 0.1 M sodium cacodylate solution (Sigma), followed by storage at 4°C until further use. The IVDs were dehydrated in serial diluted ethanol solutions (50%–99%) and processed by critical point drying (sputtering‐coating with Au/Pd thin film using the SPI Module Sputter Coater equipment). SEM was performed at the Materials Centre of the University of Porto (CEMUP), using a High resolution Scanning Electron Microscope with X‐Ray Microanalysis—JEOL JSM 6301F/Oxford INCA Energy 350—at 300× and 5000× magnification.

### 2.2 Bovine nucleus pulposus (bNP) cells isolation

Coccygeal IVDs from young (12‐month‐old) and old (10 to 16 years old) bovine animals (male) were dissected until 3 h after death. The NP was harvested from 7 to 8 IVDs from the same animal, and cells were isolated according to a previously established protocol (Molinos et al., 2015). Briefly, NP tissue was separated from AF and vertebral endplates with a scalpel blade, and finely chopped. Tissue digestion was performed in Dulbeccos's modified Eagle's medium (DMEM, 21885; Gibco), supplemented with 5% v/v Penicillin Streptomycin (PAA), 10% v/v Amphotericin B. (PAA), 2.5% (v/v) of HEPES Buffer 1 mM (Lonza), 1.5% (v/v) of NaCl 5 M and KCl 0.4 M solution (to adjust osmolarity to 400 mOsm), 1.3 U/mL DNAse and collagenase‐type‐XI (Coll‐XI) (C7657; Sigma Aldrich), at 2.0 mg/mL. Tissue/medium ratio was set at 10% w/v to prevent the pH from dropping below 6.8 during incubation. Tissue was digested for 4–5 h (until complete digestion) in a humidified atmosphere at 37°C/5% CO_2_, and under gentle stirring. Finally, ECM contaminants were removed with a 70 μm cell strainer, and cells were resuspended in 10 mL DMEM supplemented with 5% FBS, 1% v/v Penicillin Streptomycin, 0.5% v/v Amphotericin B, and osmolarity adjusted to 400 mOsm.

### 2.3 Gene expression of bNP cells by quantitative real‐time reverse transcription–polymerase chain reaction (qRT‐PCR)

Gene expression levels were determined by quantitative real‐time reverse transcription–polymerase chain reaction (qRT‐PCR) as described by Teixeira et al. with slight modifications (Teixeira et al., 2016). Briefly, for RNA isolation, NP samples were digested enzymatically, as described above, and cell pellets were recovered by centrifugation at 400 *g* for 10 min. Total RNA was then extracted from NP cells using ReliaPrep RNA Cell Miniprep System (Promega), according to manufacturer's instructions. Total RNA was quantified using a NanoDrop 1000 spectrophotometer (Thermo Fisher Scientific). Finally, isolated RNA preparations were pre‐treated with Turbo DNA‐free kit (AM1907; Ambion, Invitrogen) to remove contaminating DNA, and posteriorly the RNA was reverse transcribed into cDNA using SuperScript III Reverse Transcriptase (18080‐093; Invitrogen) as described by the manufacturer. The cDNA from bNP cells was diluted at a ratio of 1:4 in RNase‐free water (AM9938, Ambion, Invitrogen) and used for qRT‐PCR. Specific primer sets for bovine glyceraldehyde 3‐phosphate dehydrogenase (GAPDH), collagen type‐II, aggrecan, IL‐6, IL‐8, MMP1, and MMP3 (Table 1) were designed using published gene sequences (PubMed, NCBI Entrez Nucleotide Database) and Primer3 software, and purchased from Thermo Fisher Scientific. Real‐time quantitative polymerase chain reactions (PCR) were conducted on a iQ5 Real‐ Time PCR Detection System (Biorad), and performed according to the SYBR Green method, in triplicate, in PCR 96‐well TW‐MT‐Plates (Biozym Scientific), under standard conditions. Reaction mixes contained 12.5 mL of Platinum SYBR Green qPCR SuperMix‐UDG (Invitrogen) master mix, 0.25 mL of ROX Reference Dye (Invitrogen), 1 mL (0.4 mM) of forward primer, 1 mL (0.4 mM) of reverse primer, 8.25 m L of RNase‐free water, and 2 mL of cDNA. For the analysis of the mRNA expression, cloned amplification products were provided and used as standards for qRT‐PCR. Statistical analysis was performed on ΔCt values. The average Ct value of each triplicate measurement of each sample was normalized to the house‐keeping gene glyceraldehyde 3‐phosphate dehydrogenase (GAPDH) in each sample [ΔCT = CT (gene of interest) – CT (GAPDH)] (*n* = 4–6).

**TABLE 1:** Bovine primer sets used for qRT‐PCR analysis.

Gene	Sequence (forward and reverse primer)	Product length (bp)	NCBI reference sequence
GAPDH	5′ ‐ACC CAG AAG ACT GTG GAT GG‐3′ 5′ ‐CAA CAG ACA CGT TGG GAG TG‐3′	148	XM_001252511
COL2A1	5′ ‐CCT GTA GGA CCT TTG GGT CA‐3′ 5′ ‐ATA GCG CCG TTG TGT AGG AC‐3’	193	X02420
ACAN	5’ ‐ACA GCG CCT ACC AAG ACA AG‐3′ 5′ ‐ACG ATG CCT TTT ACC ACG AC‐3′	237	NM_173981
IL‐6	5′ ‐ACC CCA GGC AGA CTA CTT CT‐3′ 5′ ‐GCA TCC GTC CTT TTC CTC CA‐3′	178	EU276071
IL‐8	5′ ‐ATT CCA CAC CTT TCC ACC CC‐3′ 5′ ‐ACA ACC TTC TGC ACC CAC TT‐3′	145	AF232704
MMP‐1	5′ ‐ATG CTG TTT TCC AGA AAG GTG G‐3′ 5′ ‐TCA GGA AAC ACC TTC CAC AGA C‐3′	155	NM_174112.1
MMP‐3	5′ ‐AAT CAG TTC TGG GCC ATC AG‐3′ 5′ ‐CTC TGA TTC AAC CCC TGG AA‐3′	183	AF069642

Abbreviations: GAPDH, glyceraldehyde 3‐phosphate dehydrogenase; IL, interleukin; MMP, metalloprotease; qRT‐PCR, quantitative real‐time reverse transcription–polymerase chain reaction.

### 2.4 Sulfate GAGs quantification

Sulfate GAGs (sGAGs) quantification was performed in young and old bovine NPs, by Blyscan Sulfate GAGs Assay (biocolor) kit, as in Fiordalisi et al. (2022). Briefly, after tissue digestion in Papain Extraction Reagent, sGAGs were quantified following the manufacturer's instructions. Absorbance was measured at 656 nm at the Synergy™ Mx multi‐mode microplate reader (BioTek). SGAGs concentration was calculated by adopting a standard curve and normalized by samples' wet weight.

### 2.5 ECM staining of bNP tissue

Young and old bovine NPs were isolated from the IVDs by using 4 mm surgical punches (Kai medical). After, samples were fixed in 10% neutral buffered‐formalin (Bio‐Optica), overnight at 4°C and processed for paraffin embedding. Finally, 5 μm sections were obtained and stained for the different proteins of interests, as previously optimized (Fiordalisi et al., 2022). Briefly, for Alcian Blue and Picro‐Sirius Red staining, sections were incubated in Gill's Hematoxylin (Thermo Fisher Scientific) for 3 min. Next, a staining in Alcian Blue for 30 min followed by Picro‐Sirius Red for 1 h were performed. Images were acquired at optical microscope (Zeiss) at 10× objective.

Immunohistochemistry (IHC) for ACAN and immunofluorescence (IF) for COL2 were performed as optimized in Fiordalisi et al. (2022). Briefly, for ACAN IHC sections were incubated in 20 μg/mL Protenease K solution for 15 min at 37°C. After, IHC was conducted by using the NovolinkTM Polymer Detection Kit (Leica Biosystem), following manufacturer's instructions. An incubation with diluted primary antibody (1:750, anti‐ACAN monoclonal antibody BC‐3; MA3–16888; Invitrogen), was performed overnight at 4°C. Images were acquired at optical microscope (Zeiss), at 20× objective.

For COL2 IF, sections were immersed in boiled citrate buffer for 1 min and afterwards incubated with 20 μg/mL Protenease K solution for 15 min at 37°C. After blocking (1 h), sections were incubated with the primary antibody (dilution 1:50; anti‐COL2A1 monoclonal antibody M2139; sc‐52658, Santa Cruz Biotechnology), overnight at 4°C. Next, an incubation with a secondary antibody (dilution: 1:1000; Alexa Fluor™ 594 goat anti‐mouse; A11020; Invitrogen) for 1 h at room temperature, was performed in order to detect the protein of interest. Finally, sections were mounted with DAPI mounting media and images were acquired at Confocal Microscope (TCS, SP5; Leica Microsystems) at 20× objective.

### 2.6 Proteomic analysis

We re‐analysed the data of the NP proteomic profile obtained by us (Caldeira et al., 2017), focusing only on the NP cellulome by removing all the proteins from the matrisome (a comprehensive list of genes coding for ECM molecules and regulators). By doing so, we ended up with 53% of proteins exclusively associated with the NP cell signature. We defined the NP cellulome as the 41 cellular proteins identified in the 2 age groups (relative protein quantification of such molecules is summarized in Figure 2c). Results (from at least three independent biological samples) were expressed as mean ± SEM Value, *p* ≤ 0.05 was considered statistically significant. To explore the types of functions affected by the proteins and corresponding genes identified, we used the Functional Annotation Clustering Tool from Database for Annotation, Visualization and Integrated Discovery (DAVID) Bioinformatics Resources version 6.8 (https://david.ncifcrf.gov/) (Huang da et al., 2009). This allowed us to determine whether particular GO terms occurred more frequently than expected by chance, in a given set of genes. Statistical enrichment of GO terms was calculated using the default background set provided by DAVID. Genes were then clustered according to functional similarity. The software uses the Fisher Exact test to measure functional enrichment in annotation categories from numerous public databases. The over‐representation *p*‐value is calculated based on, EASE Score, a modified Fisher Exact Test (*n* = 3–6).

### 2.7 Flow cytometry analysis of bNP cells

The expression of surface markers was analysed in freshly isolated cells bNP cells by flow cytometry upon overnight recovery of cell surface antigens in 15 mL falcon tubes with loosely tightened caps as previously optimized in our group (Molinos et al., 2015). Firstly, cells were washed with PBS‐2% Fetal bovine Serum (FBS) and then incubated with primary antibodies (Table 2), in the same solution, for 1 h at RT. The antibodies used stained for: CD29 (integrin β1), CD44 (receptor of hyaluronic acid), CD146 (receptor for laminin alpha 4, marker for mesenchymal cells), Stro‐1 (MSCs marker), Tie‐2 (angiopoietin receptor, marker for NP cell progenitors), GD‐2 (disialoganglioside, a marker for NP cell progenitors), CD45 (pan‐hematopoietic marker), CD34 (hematopoetic progenitor or endothelial cells). After labelling, cells were washed with 2 mL PBS‐2% FBS. In the case of non‐conjugated anti‐CD34 and anti‐GD2 antibodies, cells were additionally incubated in the same conditions with a secondary donkey anti‐mouse IgG‐AlexaFluor647® antibody, and in case of anti‐Stro‐1, cells were incubated with a secondary goat anti‐mouse IgM‐FITC antibody, and then washed. Then bNP cells were incubated with Propidium Iodide (PI) (2 μg/mL) to exclude dead cells and run in a FACSCanto (BD Biosciences). Cells were stained always with PI and 2 or 3 antibodies. Gates were performed in PI negative cells based on isotype controls. Results were analysed with FlowJo software, version 8.7, as previously described (*n* = 3–11). (Molinos et al., 2015).

**TABLE 2:** Antibodies used in flow cytometry analysis of bovine NP cells.

Antibodies	Fluorochrome	Conc. (μg/mL)	Clone	Catalog # manufacturer
*Primary antibodies*				
Mouse anti‐bovine CD29	AlexaFluor®488	5 μL/10^5^ cells	TS2/16	303015, Biolegend
Mouse anti‐bovine CD44	FITC	10	IL‐A118	MCA2433F, AbD Serotec
Mouse anti‐bovine CD45	FITC	10	CC1	MCA832F, AbD Serotec
Mouse anti‐human CD146	AlexaFluor®647	5	OJ79c	MCA2141A647T, AbD Serotec
Mouse anti‐human Tie‐2	PE	5 μL/10^5^ cells	83715	FAB3131P, R&D Systems
Mouse anti‐bovine CD34	–	24		Isotype N21, kindly provided by Sakurai M {Sakurai, 2006 #231
Mouse anti‐human GD2	–	20	14.G2a	554272, BD Pharmigen
Mouse anti‐human Stro‐1	–	20	STRO‐1	14‐6688‐82, eBioscience
*Secondary antibodies*				
Goat anti‐mouse IgM	FITC	5		F0118, R&D Systems
Donkey anti‐mouse IgG	AlexaFluor®647	4		A31571, Life Technologies
*Isotype controls*				
Mouse IgG1, κ	AlexaFluor®488	20	MOPC‐21	400132, Biolegend
Mouse IgG1	FITC/R‐PE	5 μL/10^5^ cells		M1FP, Life Technologies
Mouse IgG1	AlexaFluor®647	5		sc‐24636, Santa Cruz Biotech
Mouse IgG1 k purified	–	20	P3.6.2.8.1	14‐4714, eBioscience
Mouse IgG2a, κ purified	–	20	G155‐178	556651, BD Pharmigen
Mouse IgM purified	–	20	PFR‐03	21275051, Immunotools

### 2.8 Imaging flow cytometry (IFC) analysis of bNP cells

To go further on bNP cell characterization, we examined the morphological features of young and old bNP cells by IFC, as previously performed (Molinos et al., 2018). For that bNP cells were fixed in PFA 4% at RT for 15 min and washed with PBS. Shortly prior to acquisition, cells were labelled with DRAQ5 (65‐0880; eBioscience), and run on ImageStreamX (IS, Millipore). Analysis was performed for morphological features (single cell events, multinucleated events, cell area, cell diameter, percentage of cells containing bright vesicles, and mean number of bright vesicles per cell) as previously described (*n* = 3) (Molinos et al., 2015).

### 2.9 Statistical analysis

Statistical analysis was performed using GraphPad Prism version 9.0 for Mac OS. D'Agostino and Pearson omnibus normality test was used to assess Gaussian distribution of data. Comparison between gene expression and cell phenotype of young vs old NP cells followed a non‐parametric distribution and were analysed by the unpaired Mann–Whitney test. Proteomics statistical analysis was performed using standard t‐test from Markerview Software. The flow cytometry data regarding the comparison between the different cell populations in young vs old NP cells were analysed by two‐way ANOVA, with a Sidak multiple comparison's test. In all cases, a confidence level of at least 95% (*p* < 0.05) was considered.

## 3 RESULTS

### 3.1 Gene expression and proteomic profiling of bNP cells with aging

**FIGURE 1:**
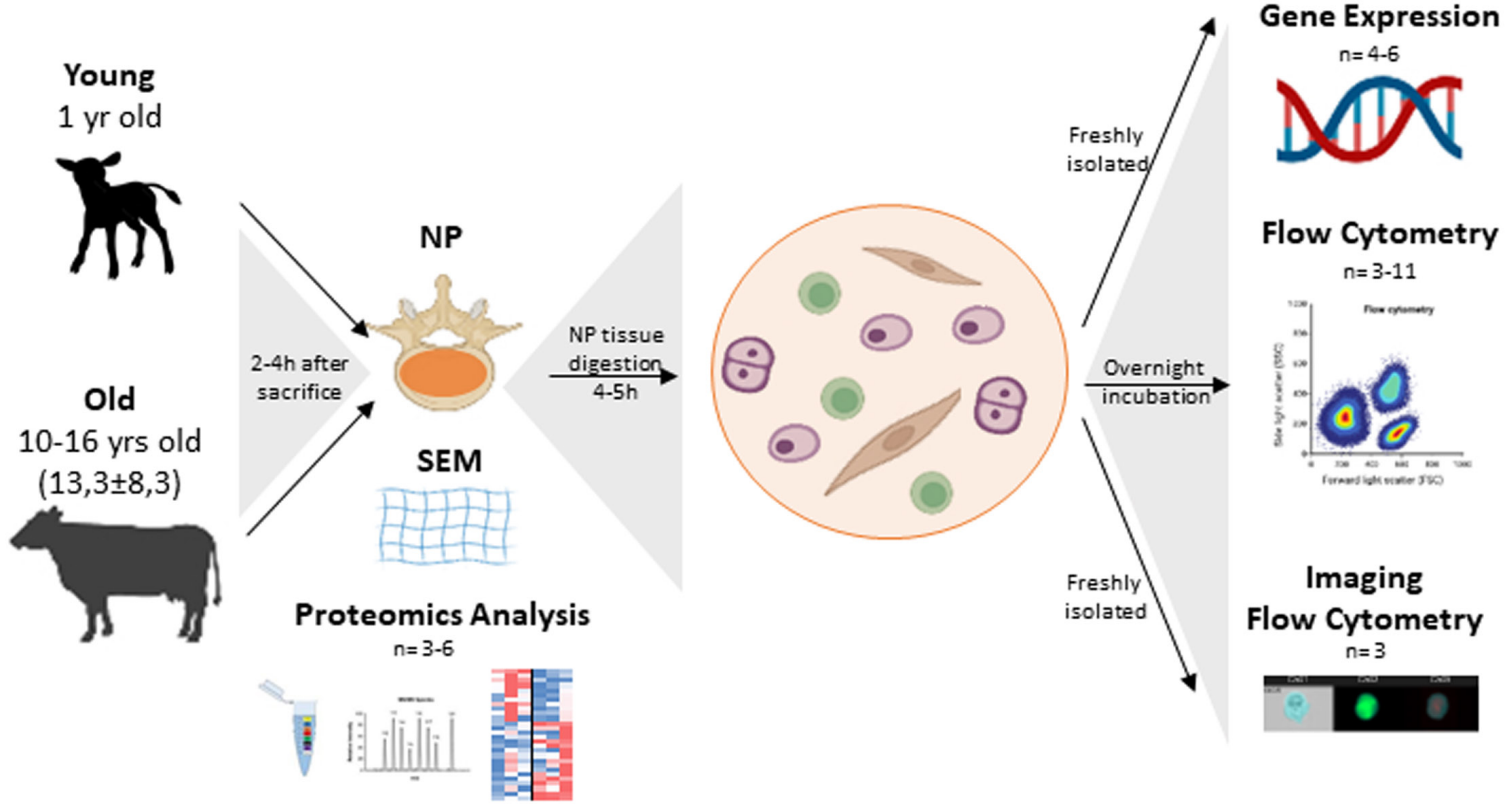
Schematic representation of the isolation and characterization of bNP cells from young and old animals.

**FIGURE 2:**
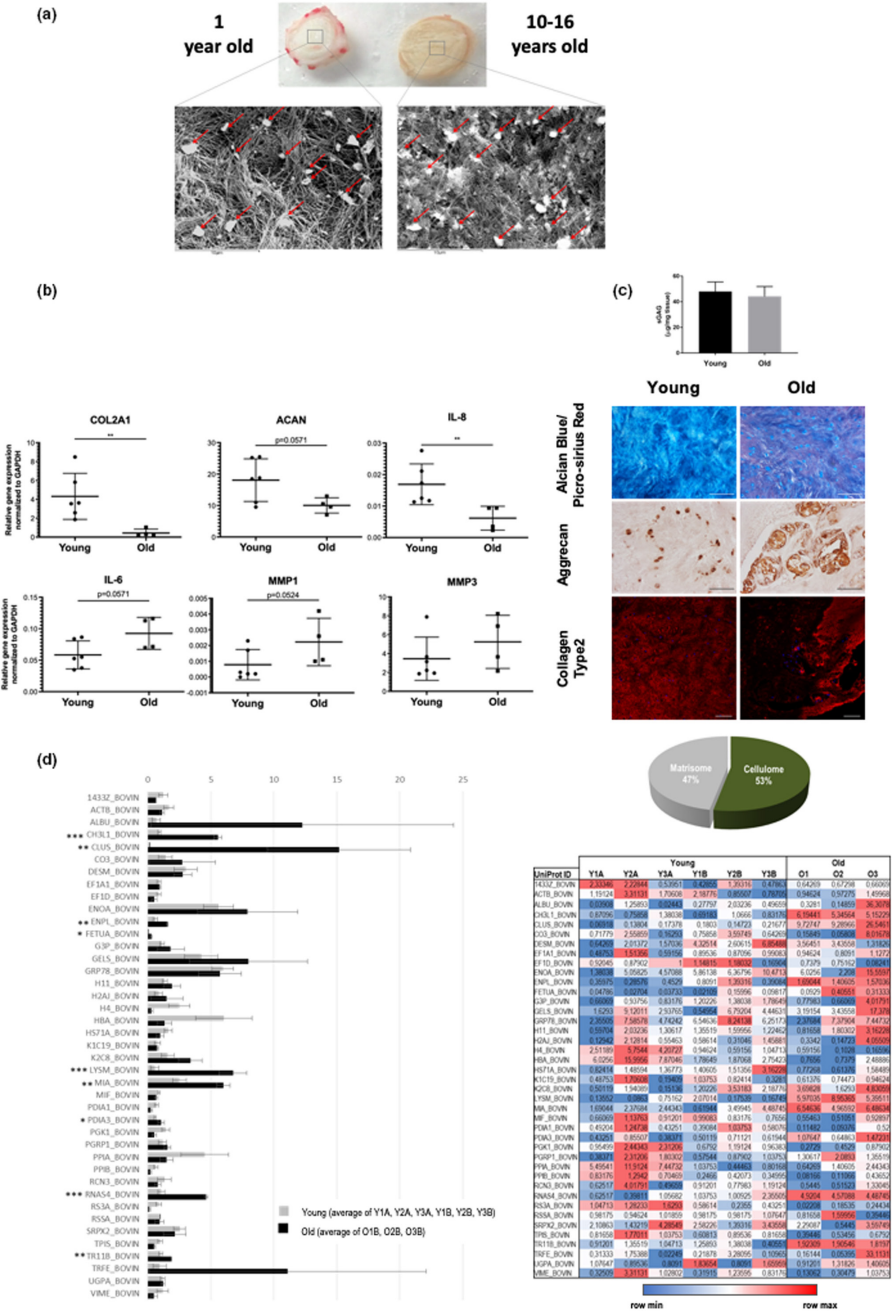
(a) Scanning electron microscopic (SEM) image of young (1‐year old) and old (10–16 years old) bovine NP. (b) Gene expression of cells freshly isolated from young and old bovine NPs. Relative mRNA expression of ECM components (Coll II and Agg), pro‐inflammatory markers (IL‐8 and IL6) and matrix degrading enzymes (MMP‐1 and ‐3), normalized to the housekeeping gene GAPDH. Results are presented by points with Mean ± StDev (*n* = 4–6, **p < 0.01, Mann–Whitney test). (c) Sulfate GAGs (sGAGs) quantification by Blyscan Sulfate GAGs Assay in young and old bovine NPs. Data are normalized by wet weight and reported as μg sGAGs/mg wet weight. Results are presented with Mean ± StDev (*n* = 3, Mann–Whitney test). Alcian Blue and Picro‐Sirius Red staining of young and old NPs. Red: collagen, blue: GAGs, purple: cell nuclei. Magnification 10× and scale bar 200 μm. ACAN Immunohistochemistry (IHC) and COL2 immunofluorescence (IF) of young and old NPs. Magnification 20× and scale bar 100 μm. (d) NP proteomics analysis‐ The pie charts exhibit percentages of identified proteins distributed by cellulome or matrisome categories. The NP cellulome signature of young vs aged bovines was compared. iTRAQrelative protein expression levels (*x* axis) are displayed for each of the molecules identified (*y* axis) (Young ‐grey vsOld – black). iTRAQ protein quantification scores for individual samples are in the table (colour scale from blue – low expression – to red – high expression). In the case of Young animals, the values represent an average of the technical replicates. Standard *T*‐test performed in Markerview was used to compare the two groups of non‐related samples. *n* = 3–6, standard error of the mean (SEM) is represented as the error bar. (*) stands for *p* ≤ 0.05 and (**) for *p* ≤ 0.01.

To dissect the differences in bNP cells with aging, bovine NPs from animals with 1 year old (young) and more than 10 years old (old) were collected and analysed through different techniques (Graphical Abstract, Figure 1). At the macroscopic level, the white and gel‐like NP becomes yellowish and stiffer, with increased frequency of calcium deposits as suggested by SEM observations (Figure 2a), and confirmed by Alizarin Red staining (Figure S1). In what concerns gene expression differences of bNP cells, we have analysed gene expression of pro‐inflammatory markers, MMPs and ECM proteins that were previously addressed in the establishment of a standardized bovine NP organ culture under pro‐inflammatory/degenerative conditions (Teixeira et al., 2016). This panel of gene expression markers was used due to the strong similarities between disc degeneration and disc aging, such as structural failure or water loss (Galbusera et al., 2014).

Relatively to the alterations of these cells with aging, bCOL2A1, bACAN and bIL‐8 were reduced (*p* < 0.01 for COL2A1 and IL‐8, and *p* = 0.0571 for ACAN), while bIL‐6 and bMMP‐1 showed a tendency to increase (*p* = 0.0571 and *p* = 0.0524, respectively) (Figure 2b). No clear differences were observed in bMMP‐3 gene expression. To confirm ECM gene expression, sGAGs and Collagens were analyzed at the protein level (Figure 2c). Similar sGAGs content was observed, with a tendency to slightly decrease with aging. Aggrecan is equally expressed in young and old NP cells, being evident the staining in cells clusters with aging. Regarding collagens, we observed an increase of red staining (by picro‐sirius red) with aging and a reduction of collagen type 2 by immunofluorescence, suggesting that red staining might be collagen type 1, that is increasing with aging. The apparent reduction in collagen type 2 is in agreement with gene expression data.

By reanalysing the NP proteomic data obtained by Caldeira et al. (2017), we focused our attention on the cellular proteins. As so, we defined the NP cellulome as 41 non‐matrisomal proteins, identified independently of the different age groups. These proteins represent 53% of the entire NP signature (Figure 2d). To explore the biological processes affected by these 41 cellulome‐associated proteins commonly identified in all of the native NP samples, and thus related to NP cellular changes with aging, we performed Gene Ontology (GO) term enrichment analysis using the Functional Annotation Clustering Tool from DAVID Database. This allowed us to determine GO terms that occurred more frequently than expected by chance. Proteins were then clustered according to functional similarity (Table S1). The most significant cluster of proteins, in comparison to *Bos taurus* proteome, included GO terms implicated in endoplasmic reticulum and melanosome.

For the identification of potential NP age‐related cellular components, we considered those proteins with a protein expression ratio cut‐off >1.5 and <0.67 and a statistically significant difference. No cellular proteins were identified as being upregulated in young NPs in comparison with old NPs. In contrast, a few of them were overexpressed in aged NPs, namely Chitinase‐3‐like protein 1, Clusterin, Endoplasmin, Alpha‐2‐HS‐glycoprotein, Lysozyme C, Melanoma‐derived growth regulatory protein, Protein disulfide‐isomerase A3, Ribonuclease 4 and Tumour necrosis factor receptor superfamily member 11B (Figure 2d). Interestingly, when inquiring on their functional significance, we found that there was an enrichment in proteins implicated in dissulfide bonds, glycoproteins and glycosylation, and once again, in the endoplasmic reticulum among others (Table S2).

### 3.2 Phenotype of bNP cells with aging

**FIGURE 3:**
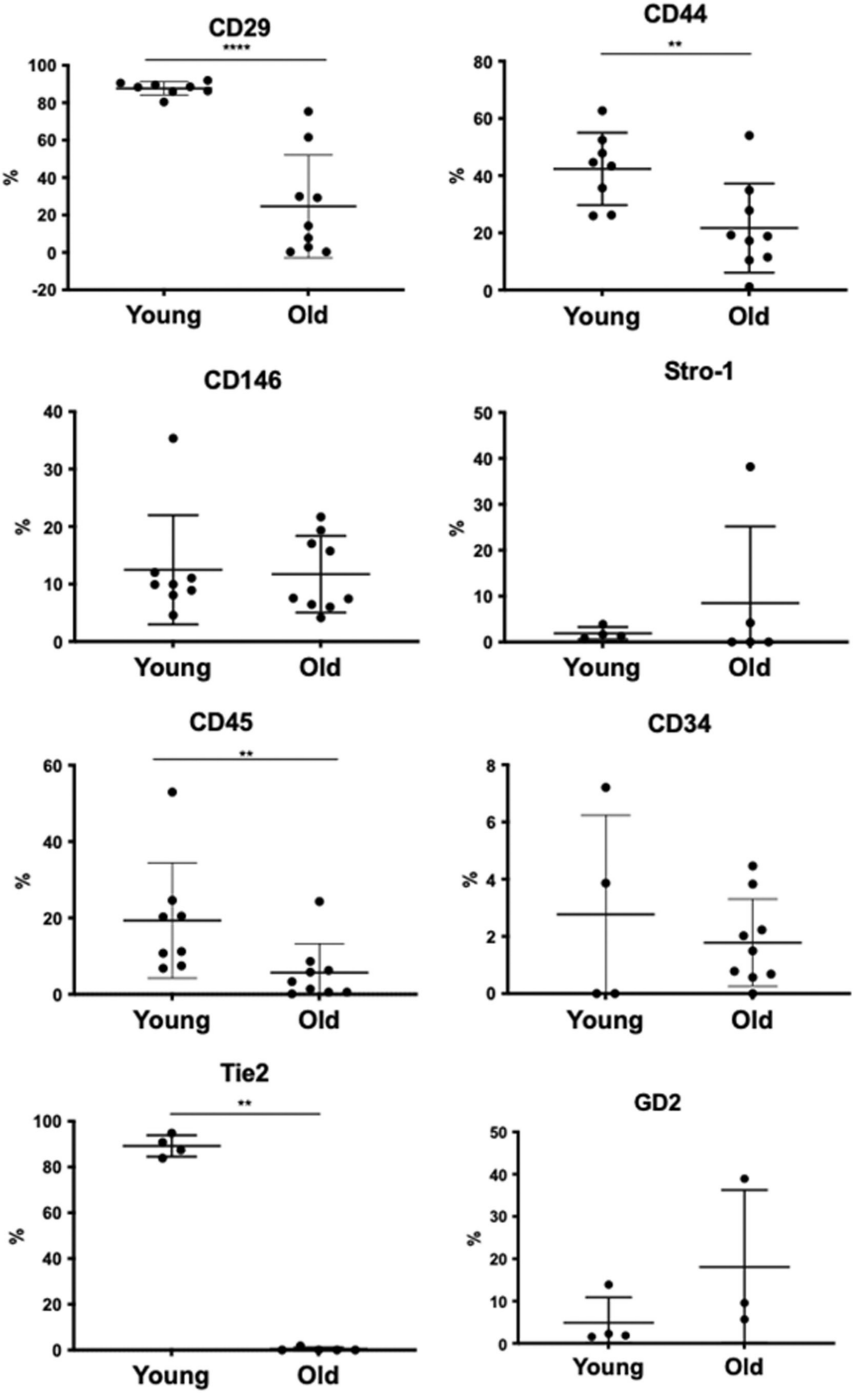
Immunophenotypic profile of bNP cells freshly isolated from young and old animals. Percentage of live cells expressing the surface markers CD29, CD44, CD146, Stro‐1, CD45, CD34, Tie2, GD‐2, after subtracting the respective isotype control. Results are presented as points with Mean ± StDev (*n* = 4–8 for young, *n* = 3–9 for old, **, *p* < 0.01, ****, *p* < 0.0001, Mann–Whitney test).

To assess the effect of aging on bNP cells surface markers signature, a panel of 8 markers was selected, 5 of which (CD29, CD44, CD45, CD146 and CD34) had been described in our previous work characterizing bNP cells from young animals (Molinos et al., 2015). Here we have added three additional bovine surface markers (Figure 3): (i) Stro‐1 (a marker for MSCs subpopulation); (ii) Tie‐2 (angiopoietin receptor, a marker for NP cell progenitors); and (iii) GD2 (disialoganglioside, a marker for MSCs, that has been already described in NP, particularly for NP progenitor cells) (Sakai et al., 2012). The plots and gates used to quantify the percentage of positive cells for each marker in young and old bovine animals are presented in Figure S2. We observed that young and healthy bNP cells have a high percentage of cells expressing CD29 (88 ± 4%), CD44 (42 ± 13%) and Tie2 (89 ± 5%), intermediate amounts of CD45+ (19 ± 15%) and CD146+ (13 ± 10%) cells and low percentages of CD34+ (3 ± 3%), Stro‐1+ (2 ± 1%) and GD2+ (5 ± 6%) cells. Along aging, there was a significant decrease in the percentage of bNP cells expressing CD29 (26 ± 27%, *p* < 0.0001), CD44 (22 ± 16%, *p* < 0.01) or Tie2 (0.4 ± 0.8%, *p* < 0.0001), while the percentage of cells expressing CD146 and CD34 was maintained, and there was a slight increase in Stro‐1+ (8 ± 16%) and GD2+ (18 ± 18%) cells (Figure 3).

### 3.3 Identification of bNP cells subpopulations by flow cytometry

**FIGURE 4:**
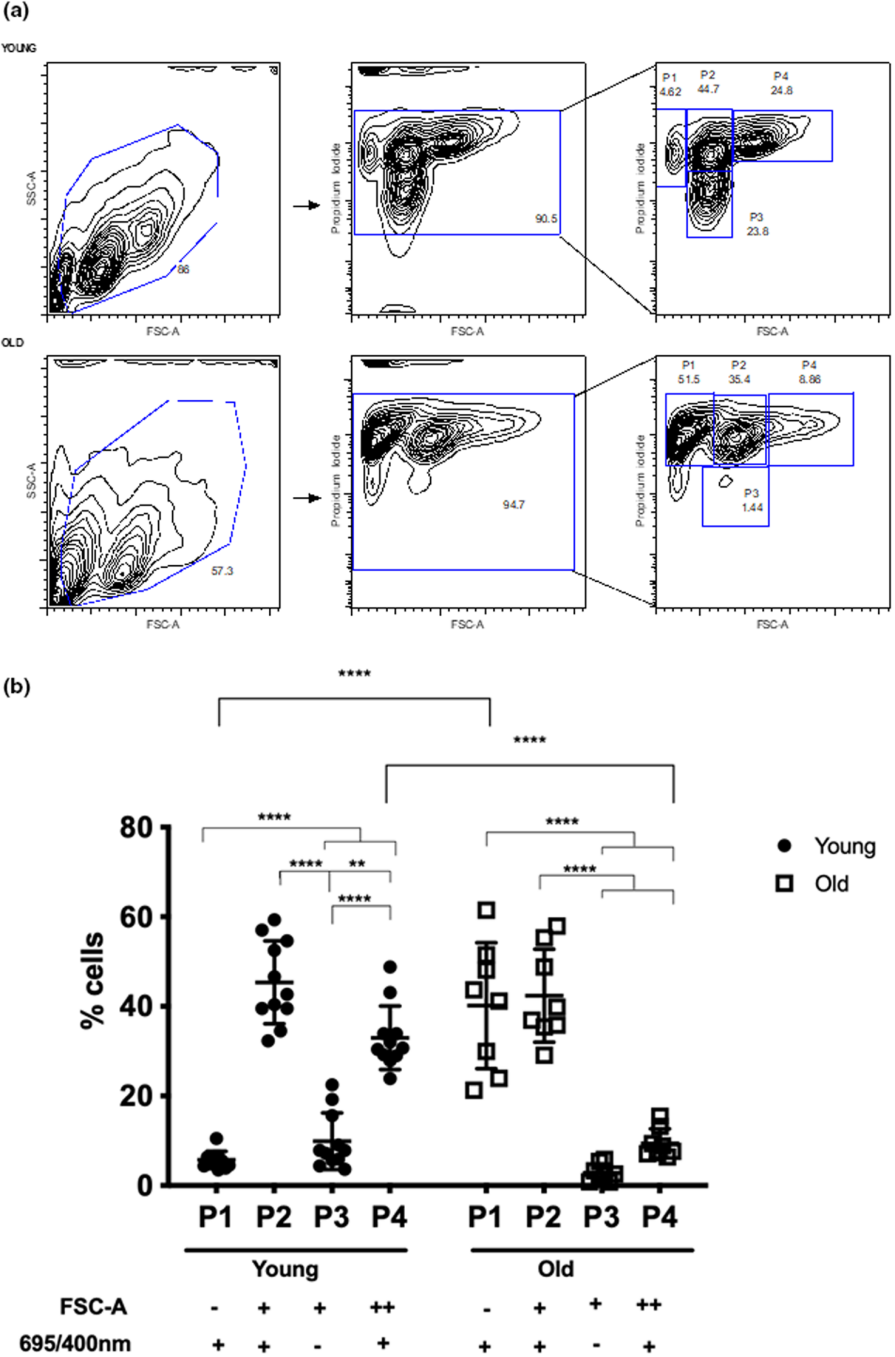
Flow cytometry analysis of cell area and auto‐fluorescence of live bNP cells from animals with distinct ages. (a) Representative flow cytometry contour plots of size and auto‐fluorescence of live (PI negative) bNP cells from young (top) and old (bottom) animals, identifying 4 sub‐populations (P1, P2, P3, P4). (b) Frequency of live (PI negative) bNP cells sub‐populations (P1, P2, P3, P4) with distinct size and auto‐fluorescence. Results are presented as points with Mean ± StDev (*n* = 15 and 11 for young and old animals, respectively, **, *p* < 0.01, ****, *p* < 0.0001, Two‐way ANOVA followed by Sidak's multiple comparison test).

To further dissect bNP subpopulations, samples were also labelled with PI (to discard dead cells, PI positive) and then analysed by flow cytometry in a FACSCanto concerning their size (FSC‐A), as previously performed in a FACSCalibur for young bNP cells (Molinos et al., 2015). After discarding dead cells and when plotting PI fluorescence intensity (channel 695/40 nm) vs FSC‐A, four subpopulations of cells with distinct areas and auto‐fluorescence wereconsistently identified in both young and old bNP cells (named P1, P2, P3, P4) (Figure 4a). Using FSC‐A in the FACSCanto allowed for a better discrimination in the FSC channel, thus allowing for the clear distinction of 4 subpopulations, as opposed to the previously described 3 populations found when using a FACSCalibur and plotting for FSC‐H (Figure S3) (Molinos et al., 2015).

The four populations of bNP cells here identified in young and healthy animals consist in: P1, a population with the smallest size (low FSC‐A) and high auto‐fluorescence and low number of events (6 ± 2%); P2, a population with medium‐size events with high auto‐fluorescence, with the highest number of events (45 ± 9%); P3, containing medium‐sized events with low auto‐fluorescence (10 ± 6%); and P4, with larger‐size events and high auto‐fluorescence (33 ± 7%) (Figure 3b). Upon aging, the distribution of bNP cells in these sub‐populations changed. Notably, the subpopulation P1, i.e. bNP cells with small size and autofluorescence, significantly increased (to 40 ± 14%, *p* < 0.0001), being almost the most frequent subpopulation in old bovine animals, while P4, i.e. bNP cells with the largest size and auto‐fluorescence, significantly decreased (to 9 ± 3, *p* < 0.0001). The frequency of the other 2 populations, P2 and P3 was maintained (to 42 ± 10% and 3 ± 2%, respectively) (Figure 4b).

### 3.4 Phenotype of bNP cells subpopulations with aging

**FIGURE 5:**
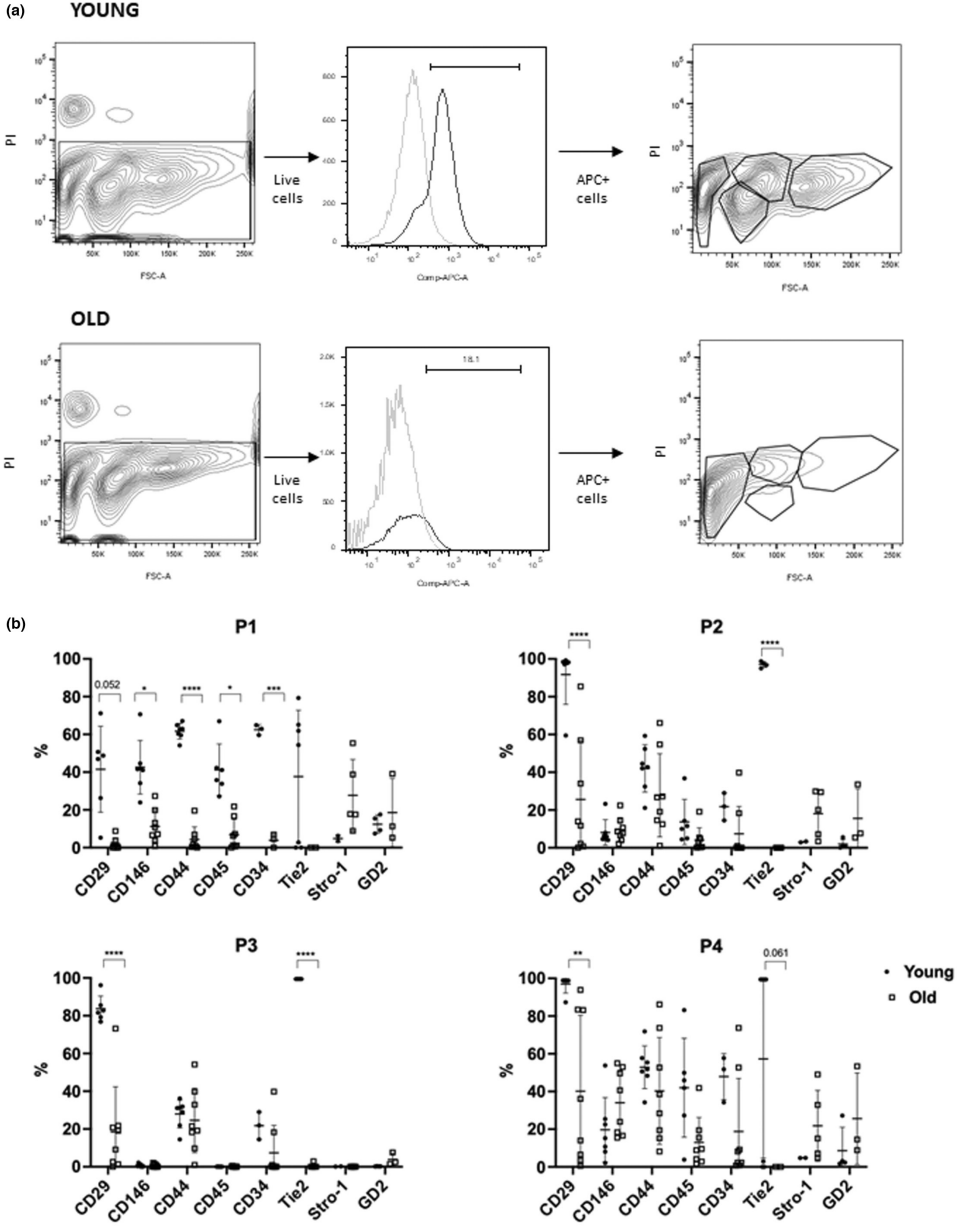
Phenotypic analysis of bNP cell subpopulations based on flow cytometry analysis. (a) Illustrative flow cytometry gating strategy used to quantify the expression of each surface marker within each subset of young and old bNP cells. (b) Percentage of cells expressing different surface markers within different bNP cells sub‐populations (P1, P2, P3, P4). Results are presented as points with Mean ± StDev (*n* = 4–8 for young, *n* = 3–9 for old, *, *p* < 0.05; **, *p* < 0.01; ***, *p* < 0.005; ****, *p* < 0.0001; Two‐way ANOVA followed by Sidak's multiple comparison test).

To better understand the features of bNP cells, we combined the phenotypic and morphological analysis of bNP cells and we investigated the alterations of the surface markers within each subpopulation of bNP cells. For that we gated live cells (PI−), followed by gating the cells positive for each surface marker (CD29, CD146, CD44, CD45, CD34, Tie2, Stro‐1, GD2). Then we analysed the distribution of the positive cells in each cell subset (P1–P4) (Figure 5a), which allowed the tracing of a phenotypic signature of each bovine NP cell subset.

Importantly, in samples from older animals, there was a significant decrease in the percentage of cells expressing CD29 (from 42 ± 23% to 1.9 ± 3.1%, *p* = 0.052), CD146 (from 43 ± 14% to 11 ± 9%, *p* < 0.05), CD44 (from 62 ± 4% to 4 ± 7%, *, *p* < 0.001), CD45 (from 41 ± 14% to 7 ± 8%, *p* < 0.05), and CD34 (from 63 ± 3% to 4 ± 4%, *p* < 0.005) within the P1 bNP cell subset. On the other hand, Stro‐1 and GD2 seemed to increase with aging but with high variability among the old donors, suggesting these are not reliable markers for IVD aging (Figure 5b).

Moreover, aging also correlated with a decrease in the percentage of cells expressing CD29 or Tie2 in the subpopulations P2, P3 and P4. CD29 significantly decreased from 92 ± 16% to 26 ± 31% in P2 (*p* < 0.001), from 84 ± 7% to 19 ± 24% in P3 (*p* < 0.001), and from 97 ± 5% to 40 ± 40% in P4 (*p* < 0.01), while Tie2 expression disappeared in all bNP subsets. A trend for an increased percentage of Stro‐1+ or GD2+ cells in P2 and P4 and of CD146+ cells in P4 with aging were observed, but without significance (Figure 5b).

### 3.5 Morphological analysis of bNP cells in flow with aging

**FIGURE 6:**
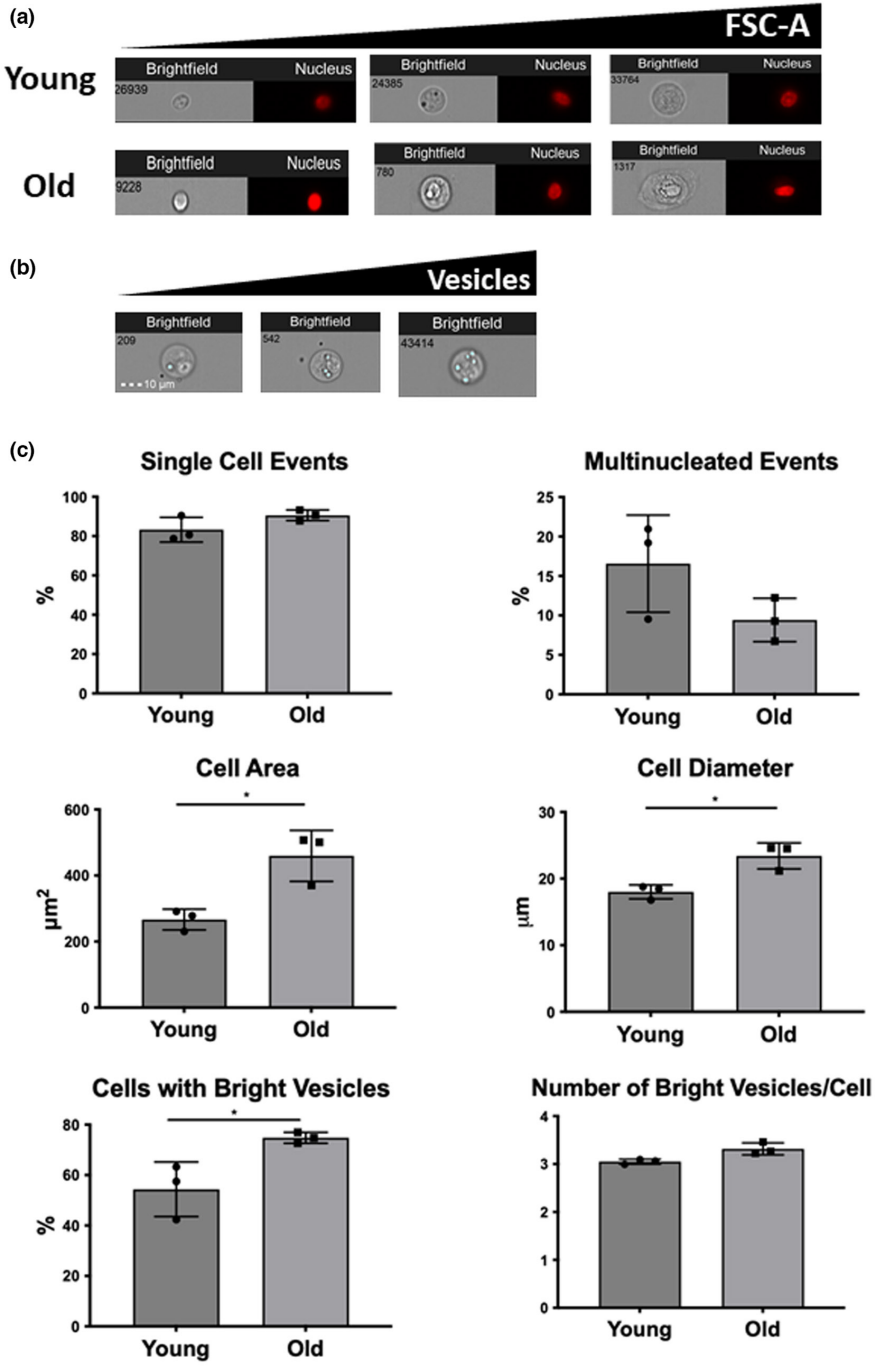
Morphology analysis of bNP cells from young and old animals by imaging flow cytometry. (a) Representative pictures of brightfield and DRAQ5 stained images of bNP cells. (b) Representative images of brightfield pictures of bNP cells with the mask used to analyse bright vesicles (light blue). (c) Quantification of the prevalence of: single cell events, multinucleated events, cell area, cell diameter, percentage of cells containing bright vesicles and mean number of bright vesicles per cell from bNP cells isolated from young and old animals. Results are presented in points with Mean ± StDev (*n* = 3, *n* = 17,994 (Y) and *n* = 2572 (O) DRAQ5+ events; *p < 0.05; Mann‐Witney test).

To go further on characterization of bNP cells using a high‐throughput method, morphological changes of bNP cells from young and old animals were also assessed using IFC. For that nucleated bNP events from young and old animals were imaged (representative images in Figure 6a). A parallel analysis of bNP cells subpopulations with conventional flow cytometry and IFC was not possible due to the lack of distinction between cell subpopulations based on cellular auto‐fluorescence (P2 and P3) by IFC (data not shown).

Morphological analysis of bNP cells revealed a similar number of single nucleated and multi‐nucleated cells with age, with significantly increased size (both in terms of cell diameter and cell area, *p* < 0.05). Also, in terms of the percentage of events containing bright vesicles (representative images in Figure 6b), these increased significantly with aging, even though the number of vesicles per cells remained constant (Figure 6c).

## 4 DISCUSSION

Our group has previously addressed the aging of bovine IVDs at the matrix level, disclosing that Collagen type XII and XIV were highly present in foetal bovine NP, while Fibronectin and Prolargin were more abundant in the elder NPs (Caldeira et al., 2017). Notwithstanding, the phenotypic profile of IVD cells with aging has been poorly studied. This work aimed at pin pointing age‐related changes in bovine NP cells using a high‐throughput method as flow cytometry, in order to identify novel targets for IVD anti‐aging/degeneration therapies. Due to the difficulties in having robust antibodies raised against bovine proteins, it is somehow tricky to use flow cytometry in its whole potential to characterize IVD cells. Still, we were able to analyze bovine NP cells surface expression of CD29, CD146, CD34, CD44, CD45, GD2, Stro‐1 and Tie2 and evaluate their alterations with age.

First, we confirmed alterations of gene expression of bNP cells with aging. Particularly, bCOL2A1, bACAN and bIL‐8 were significantly reduced, while bIL‐6 and bMMP‐1 were increased and no differences were observed in bMMP‐3. The decrease in native NP ECM proteins with aging has been largely reported in the literature, including in our previous work from bovine NP matrissome characterization by proteomics (Caldeira et al., 2017). Concerning inflammatory and proteolytic markers, IL‐6 and IL‐8 presented a contradictory trend with aging, as well as MMP‐1 and MMP‐3. These MMPs have also shown to be up‐regulated differently in human degenerated discs: MMP‐1 and MMP‐3 have been shown to increase with aging in lumbar discs, while in cervical only MMP‐3 is increased, suggesting a different pattern between both MMPs (Baptista et al., 2020). Also, a previous correlation with increased IL‐6 and IVD aging has been observed, while no apparent correlation with IL‐8 and age has been observed in the same study (Sadowska et al., 2018). Despite the extensive use of bovine IVD as a model in the field there is a lack of studies using animals with increased age, which hinders the comparison of our results with those from the literature.

Here, we found a decrease in the percentage of CD29+ bNP cells with aging. CD29 (also known as β1‐integrin) is an anchorage protein associated with stem cell niches and cell migration via cellular adhesion mechanisms, including cell–cell /cell‐matrix adhesion (Gottschling et al., 2007). Its high prevalence (72%–94%) was previously reported in human degenerated NP cells in culture (age range 21–63 years old) (Bridgen et al., 2013). In another study, a decrease in CD29 expression with age was reported by Wu et al. (2018) in human NP cells from explant cultures (about 50% in >25 years old versus about 95% on <20 years old).

Expression of CD44 was also found to decrease with aging. The expression of CD44, a transmembrane protein and the hyaluronic acid receptor, has been widely reported in the literature, with high levels in human IVD cells from degenerated (~100%) and herniated (~80%) IVD samples, but no correlation was established with aging (Marfia et al., 2015). A high prevalence was also reported in other studies using IVD and MSC cells in culture, which can affect CD44 expression (Baustian et al., 2015; van den Akker et al., 2020). CD44 expression was also assessed by Wu et al. (2018), who reported values in the range of 95% for IVD cells in culture. On the other hand, Stevens et al. reported a decrease in CD44 expression in rat NP cells associated with animals aging and loss of notochordal cell populations (Stevens et al., 2000), using immunohistochemistry, which is closer to our own observations.

Regarding CD45 prevalence, it was verified its expression was highly variable in young bNP cells in this study (1.5%–36%). The expression of an hematopoietic cell marker was somehow unexpected, since the IVD is only peripherally irrigated and we avoid blood contamination during cell isolation. Still, CD45 had been previously detected by flow cytometry in human IVD (~10%–15%) from both degenerated (age range 26–79 years old) and herniated (age range 25–56 years old) tissue, after cell expansion in 2D culture (Marfia et al., 2015). Nevertheless, in a previous study from our group using freshly isolated human IVD cells from degenerated discs, we could not find CD45 expression (Molinos et al., 2018), but samples from patients with increased aging were used (age range 43–69 years old).

The Tie‐2 expression was also analysed, based on Sakai et al. (2012) previous report, stating that nearly 90% of expanded NP cells from young human samples (20 years old) expressed Tie‐2. This marker, more known as one of the receptors of the pro‐angiogenic molecule Angiopoietin, was also considered a NP‐specific progenitor cell marker for young and healthy NP cells (Risbud et al., 2015) . Sakai and colleagues found that Tie‐2 expression dropped dramatically (<10%) in donors older than 30 years old. Accordingly, in our previous study Tie‐2 expression was not found in human NP cells from patients from 33 to 66 years old (Molinos et al., 2018). Here, we found that freshly isolated bovine NP cells present high expression of Tie‐2 in young animals (~85%), that disappear with aging. In the literature, Tie‐2^+^ bovine NP cells were reported to be around 8%–12% (Sakai et al., 2018; Tekari et al., 2016). Tie‐2+ bovine NP cells obtained upon cell sorting demonstrated to be clonogenic cells, giving rise to colony‐forming units (CFU‐Fs) both, fibroblastic (CFU‐F) and spherical (CFU‐S) in semi‐solid medium (Sakai et al., 2018). Our results show a much higher expression of Tie‐2 in bovine cells, most probably due to the use of different antibodies, but more in line with the data from human IVD. Sakai et al. (2012) has shown reduction of Tie‐2 with aging, from 71% in 19 years old to 35% in 26 years old, 5% with 37 years old, reaching 0.3% at 60 years old. Tie‐2 analysis has been performed with antibodies from distinct clones (Sakai et al., 2018). In young bovine NP cells Tie‐2 has been described to be less than 4% (with a monoclonal anti‐human antibody, clone 33.1) (Wangler et al., 2019) or up to 12% (using a polyclonal anti‐rat antibody clone bs‐1300R) (Sakai et al., 2018; Tekari et al., 2016), which is much less than the values obtained in our study (around 80%). Nevertheless, no direct comparison of the Tie‐2 expression using distinct antibodies is presented, and this process might be highly influenced by the protocol used in the different labs. For example, we performed an incubation step overnight upon tissue digestion to allow cell surface antigens recovery from the harsh enzymatic digestion, which may be crucial for some antigens. In the future it will be important to standardize the Tie‐2 analysis.

GD2, another known marker of NP cell progenitor, followed an opposite tendency to the other markers, with an increase in the percentage of GD2^+^ cells present in old bovine NPs, albeit not significant. This molecule has been identified before as a marker for bone marrow MSCs (Martinez et al., 2007; Xu et al., 2009) and umbilical cord MSCs (Xu et al., 2009). Although its expression in primary mouse and human NP cells has been reported as very low (<2%), its increase during in vitro culture was correlated with a high proliferative capability in the mouse NP (Sakai et al., 2012). GD‐2 has been pointed as a surface marker for NP progenitor cells. These cells have been described as positive for at least one marker between Tie‐2 and GD‐2. NP progenitor cells GD‐2^+^Tie‐2^−^ are described as capable of differentiating into any of adipocytes, osteocytes, chondrocytes and neurons (Sakai Daisuke et al., 2010).

Concerning CD146 expression, this was maintained with aging, which was also verified in the human IVD tissue from degenerated samples (Molinos et al., 2018). Interestingly, this marker revealed a pool of chondroprogenitors within late‐stage osteoarthritic knee joints, with high clonogenic and multi‐differentiation potential (Su et al., 2015). The stable expression of CD146 (also used as a MSC marker), along with the increase in the percentage of GD2^+^ cells (reported NP‐progenitor marker as well (Sakai et al., 2012)) with age, suggests that NP cells might be able to respond to age‐associated damage. Despite these results, further functional validation of such potential is needed.

Finally, concerning CD34 and Stro‐1, both well accepted progenitor cell markers, their expression does not seem to be affected by aging, which has also been previously described in human degenerated IVDs (<10%) (Brisby et al., 2013; Marfia et al., 2015; Navone et al., 2012). Whether or not they represent small resident cell populations with therapeutic potential is a matter to be addressed in future studies.

Profiling of the NP cellulome by reanalysis of the proteomics data published by us (Caldeira et al., 2017) has confirmed a different cellular signature for samples from young vs old NPs. Despite having used a guanidine extraction method for the enrichment of ECM proteins, more than half of the candidates obtained were cytosolic components. As so, the cellulome was defined as 41 non‐matrisomal proteins identified out of the 77 that were found in all samples independently of the age groups. Bioinformatics analysis demonstrated that the highest‐ranking functional cluster of proteins was for those involved in endoplasmic reticulum and melanosome. Indeed, melanosomes have an important role in spine morphogenesis (Ellis et al., 2013). It has been described that hyperosmotic stress (as the IVD microenvironment) results in abnormally swollen melanosomes by disturbing vesicle trafficking (Bin et al., 2014). In turn, ER stress modulation by ER‐phagy, is an adaptive mechanism to stress, namely to glucose deprivation in the IVD (Luo et al., 2022).By exploring the differentially expressed proteins between young and old NP cells, we identified several age‐related components which were significantly upregulated in old NP cells. These were implicated in glycosylation, dissulfide bonds, and once again in the ER.

Concerning glycosylation, the altered N‐glycome, may play a role in inflammation and disease progression (Joyce et al., 2021). Indeed, a particular glycosignature has been previously identified in age‐associated IVD degeneration (Collin et al., 2016). Regarding dissulfide bonds, they are known for stabilizing protein conformation. They increase mechanical stability by shifting the unfolding pathway (Manteca et al., 2017). Interestingly, the accumulation of age‐associated dissulfide bonding has been proposed as a leading event in cell aging (Taş, 1978). It may prevent fibrillization or oligomerization, or be used to pack molecular cargo for intercellular transport (Fass, 2012). ER also has a role in disc cell apoptosis and IVD degeneration (Zhao et al., 2010). Apparently, ER stress is caused by the accumulation of unfolded/misfolded proteins, which reduces synthesis, while fostering degradation (Wang, He, et al., 2021). Unfolded protein response inhibits NP cell senescence under acidic condition by activating autophagy.

To highlight a couple of the candidates identified, the enrichment of TNFRSF11B in old NP cells is in line with the increased presence of calcium deposits in elder NPs. In accordance, it had been demonstrated that this protein levels correlated significantly with IVD degeneration grade and microscopic calcification number (Rutgers et al., 2010). In rats, RANK/RANKL/OPG‐positive NP cells exist and in vitro RANKL has been shown to be upregulated by IL‐1β, while in humans RANK/RANKL/OPG‐positive IVD cells have also been described to increase with the grade of degeneration (Takegami et al., 2017). Another study of scRNA‐seq in rat IVDs revealed a cell cluster that differentially expressed RUNX2, DLX5, and SP7, suggesting that osteogenic cells exist in healthy IVDs (Gan et al., 2021). RUNX2, in turn, has been described to activate LAPTM5 expression which translocates RANKL, thus indicating that these cells could be the ones responsible for the calcium deposits observed (Chen et al., 2023). In parallel, we also observed an increase in FETUA or Alpha2‐HS glycoprotein (AHSG) in old NPs, which has been associated with higher spine Bone Mineral Density (Ix et al., 2009).

In turn, ENPL is a molecular chaperone that stabilizes and folds other proteins. It localizes to melanosomes and the ER, having been described to be increased with senescence (Kim et al., 2021; Yoo et al., 2013). In line with this, and with the observed enrichment of proteins implicated in endoplasmic reticulum and melanosome in the NP cellulome, scRNA‐seq studies in rat IVDs have identified Rab38 as a new marker of NP cells (Wang, Huang, et al., 2021), potentially involved in melanosomal transport and docking, controlling melanin production and melanosome biogenesis. Interestingly, scRNA‐seq analysis of bovine IVDs from other groups validate our results. Martina et al describe a bovine NP cell cluster with an upregulation of HIF1A and its downstream targets, reported to be involved namely in endoplasmic reticulum homeostasis (Calió et al., 2021). Panebianco and colleagues have identified novel NP markers, among which PDIA4, a gene that encodes for a protein with very similar sequence and from the same family of PDIA3 which we have shown to be downregulated in old NPs and which is relevant for transport from the ER to the cell surface. Other results from the same work are also in accordance with Martina's work and with our data, namely a cluster of NP cells expressing genes related to the ER and another with upregulation of endomembrane‐associated genes like CHI3L1. We have similarly found an enrichment in proteins implicated in the ER, as well as expression of CHI3L1 in the cellulome of young bovine NPs, which was decreased in old individuals, suggesting a loss of protection against oxidative stress‐induced catabolism (Panebianco et al., 2021).

Regarding NP cell subpopulations, we have previously addressed this heterogeneity by flow cytometry, with the consistent identification of three distinct subpopulations (P1, P2, P3) (Molinos et al., 2015). Here, by changing the analysis from FSC‐H(eight)/SSC‐H(eight) to FSC‐A(rea)/SSC‐A(rea), we were able to distinguish another subpopulation, P4, containing large‐sized and highly auto‐fluorescent events. In the area parameter vs height, it was possible to detect larger events, which is particularly important when profiling NP cells, which are many times naturally arranged in chondrons (Ciapetti et al., 2012; Sharp et al., 2009), and thus should not be discarded as doublets or clumps in flow cytometry, but rather considered a native subpopulation. Regarding subpopulation P1, which includes very small events with ~10 μm, we have previously verified that this population includes nucleated cells (with DRAQ5 expression), and should also be considered in the profile of NP cells, and not discarded as in other studies (Tekari et al., 2016). For example, the existence of very small embryonic‐like cells in adult tissues (~3–5 μm in mice and ~5–7 μm in humans) has also been showed by imaging flow cytometry analysis (Zuba‐Surma et al., 2008). Interestingly, by flow cytometry analysis a substantial decrease in subpopulations P3 and P4, and an increase in P1, in bovine NP cells were observed with aging.

Concerning bNP cells morphological characterization, we observed a significant increase in size, both in terms of cell area and diameter with aging, although this was not due to an increased content of multinucleated events. This agrees with our results from human NP cells, where the cellular size also increased with aging, mostly due to an increase in the pericellular matrix (Molinos et al., 2018). In the brightfield channel it was possible to verify that bNP cells present bright vesicles, whose frequency increases with aging, but not the number of vesicles per cell. Further studies need to be performed to understand the relevance of these vesicles in NP aging.

**FIGURE 7:**
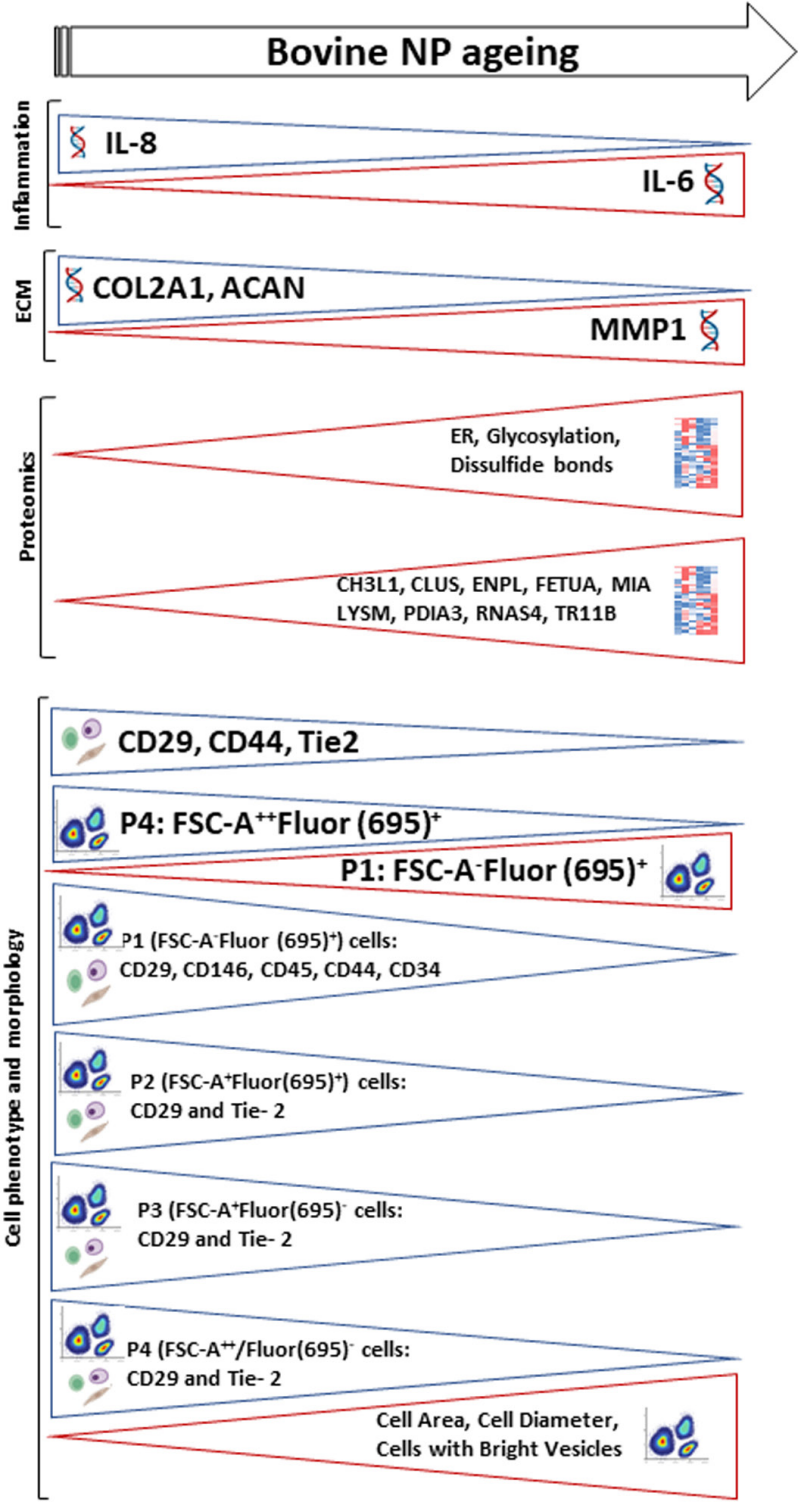
Overall scheme of the differences found on bNP cells molecular, morphological and phenotypic characteristics with aging.

Based on gene expression, proteomics, phenotypic analysis, and cell morphology we were able to improve the knowledge on NP cell aging. Moreover, by flow cytometry we were able to distinguish distinct NP cells subpopulations and their dynamics with aging (Figure 7). However, we cannot exclude this study limitations. This work was only conducted with male bovine IVDs, since these are the animals that are normally sacrificed for human consumption. The distinction between the NP cell subsets in only morphologic/phenotypic still lacks functional studies. In the future it will be crucial to sort these cell subsets and evaluate their functional role in the context of IVD degeneration. The correlation between NP cell subsets and the presence of notochordal cells was not investigated, since we lack reliable bovine markers to conduct this evaluation by flow cytometry. Furthermore, separation of bNP cell subpopulations by IFC was not possible but should be pursued in the future, with more advanced equipment with additional lasers and/or antibodies. Lastly, despite the similarities between bovine and human IVD, it would be important to validate the existence of the bNP cell subsets in human NP. However, there is a lack of access to healthy human IVD while degenerated IVD may contain infiltrated immune cells that affect this analysis.

Overall, the findings presented in this paper may better inform future therapies aiming to (1) re‐establish homeostasis, through the recapitulation of healthy NP cells signature and/or reversion of age‐dependent degenerative changes, or (2) to enhance endogenous repair by progenitor cells activation. Recapitulation of healthy NP cells signature could be achieved either by gene‐ or cell‐based approaches. Indeed, seeking to mitigate the degenerative process and also to enhance tissue regeneration, gene therapies have successfully introduced anabolic genes on IVD cells, such as growth and differentiation factor‐5, BMPs, transforming growth factor‐beta (TGF‐β) and Sox‐9 (Gazit, Kimelman‐Bleich, & Mizrahi, 2014). This strategy could also be applied to promote over expression of CD29 and CD44, two important mediators of cell‐matrix interactions and MSC markers (Henriksson et al., 2012; Knudson, 2003; Knudson & Loeser, 2002; Kurtis et al., 2001; Stevens et al., 2000), or GD2, Tie2 and CD146 progenitor cell‐related surface receptors, which may have a fundamental role in IVD repair (Martinez et al., 2007; Sakai et al., 2012; Su et al., 2015; Zhang et al., 2015). On the other hand, cell‐based approaches could enrich therapeutic cell suspensions on cells positive for those markers and repopulate the NP. Finally, activating the native regenerative cell machinery could be achieved by in situ delivery of key bioactive molecular cues (Ko et al., 2013).

## 5 CONCLUSIONS

In conclusion, our study demonstrated novel age‐associated changes in the phenotype and morphology of bNP cells. Morphologically, cell area, cell diameter and cells with vesicles increase with aging. Proteomics data was used to define the NP cellulome and how it changed with aging, unveiling an upregulation of proteins related to disulfide bonds, glycosylation and the ER. By comparing bNP cells from young and old animals, we demonstrated a decrease in the prevalence of surface markers of mesenchymal stromal cells (MSC)‐related (CD29 and CD44), hematopoietic (CD45) and NP‐progenitor (Tie‐2 and GD‐2) in bovine NP cells. Interestingly, our study revealed no alterations in the percentage of GD2^+^, CD146^+^, Stro‐1^+^ and CD34^+^ cells, supporting the idea that although the adult NP may exhibit endogenous regenerative potential, there might be an age limit in time for its activation. Moreover, morphology and auto‐fluorescence analysis of bNP cells by flow cytometry disclosed the existence of 4 subpopulations. These subsets become altered with aging, with young bNP cells presenting an increased frequency of cells with higher area and auto‐fluorescence (FSC‐A++Fluor (695)+), while cells with lower area and auto‐fluorescence (FSC‐A‐Fluor (695)+) increased with aging. In more detail, the number of cells expressing CD29, CD146, CD45, CD44 or CD34 in the subpopulation with lower area and auto‐fluorescence (FSC‐A‐Fluor (695)+) decreased with aging, while for the other NP cell subsets identified, only CD29 and Tie‐2 expression decreased. Overall, this work contributes to the characterization of NP cell aging using flow cytometry. We expect that this methodology will be replicated, contributing to advance the knowledge on the therapeutic targets for degenerative disc disease.

## AUTHOR CONTRIBUTIONS

MM has performed the experiments, collected the data and wrote the first draft of the manuscript. JC has performed the proteomics analysis and MF has performed the ECM analysis of IVD tissue. CA and RG have analysed the data and together with MB designed the experiments. All the authors have critically revised the manuscript.

## CONFLICT OF INTEREST STATEMENT

None declared.

## Supporting information


Figure S1

Figure S2

Figure S3

Table S1

Appendix S1

## Data Availability

The authors confirm that there is sufficient information for an independent researcher to reproduce all of the reported results. The proteomics data from this manuscript is accessible in a public, open‐access repository (https://doi.org/10.6019/PXD005616).
