# Supplementary figures and images for: Alterations of bovine nucleus pulposus cells with aging
Source: Aging Cell. 2023 May 30;22(8):e13873. doi: 10.1111/acel.13873 (PMC10410011; doi:10.1111/acel.13873)

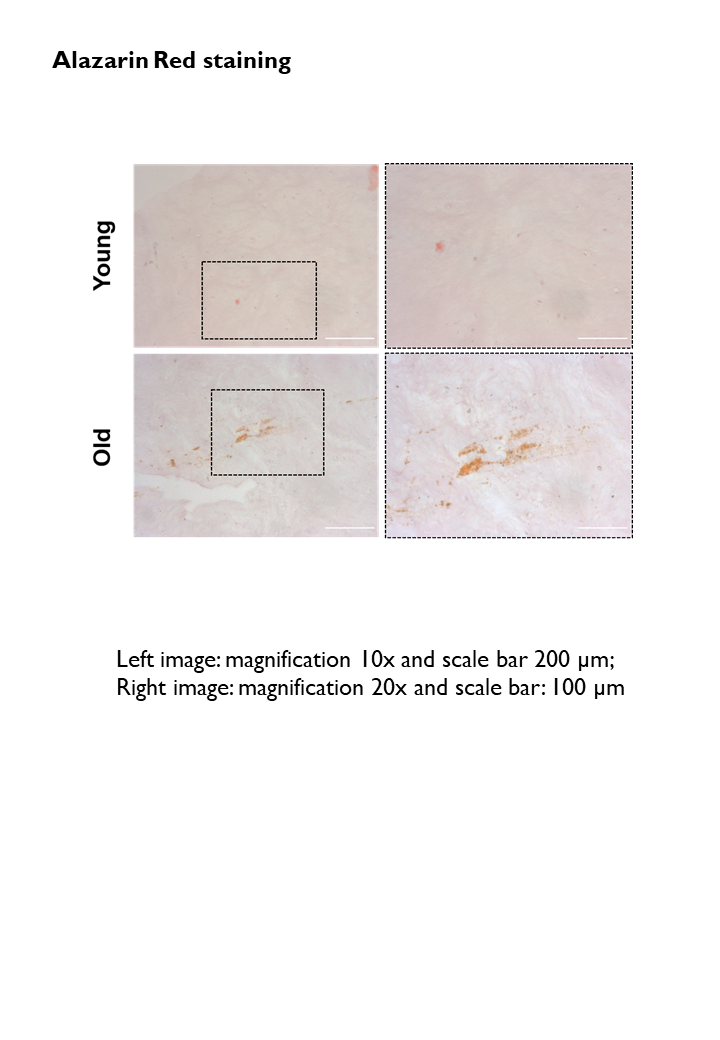

Supplement: Supplementary file 1 — Figure S1 [file ACEL-22-e13873-s001.tiff]

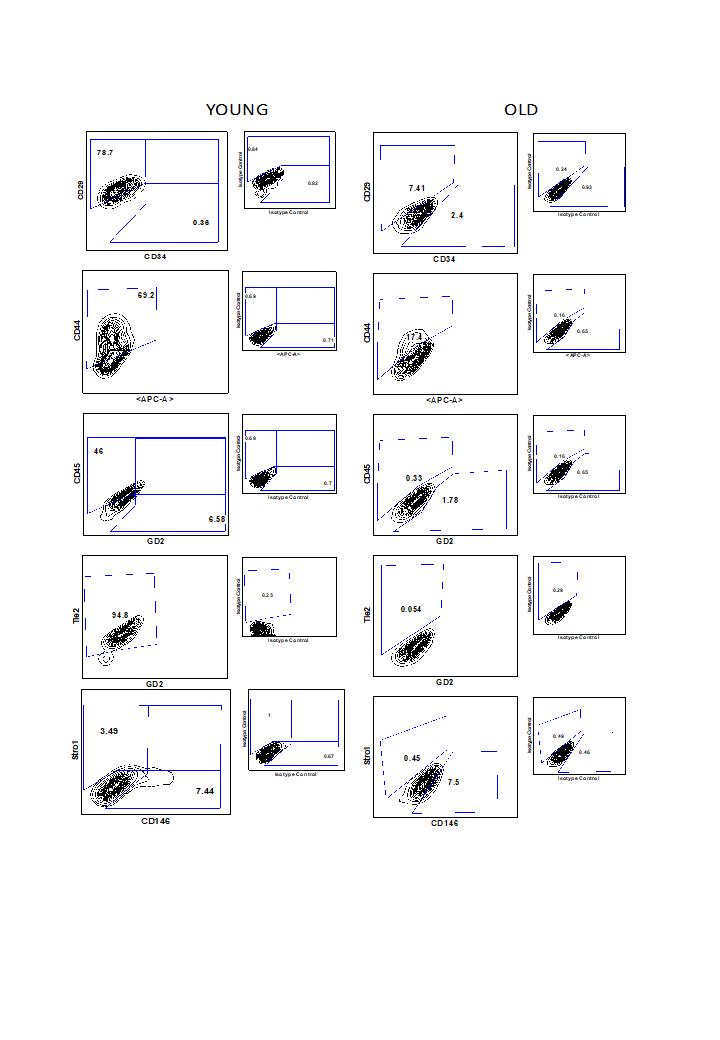

Supplement: Supplementary file 2 — Figure S2 [file ACEL-22-e13873-s003.tiff]

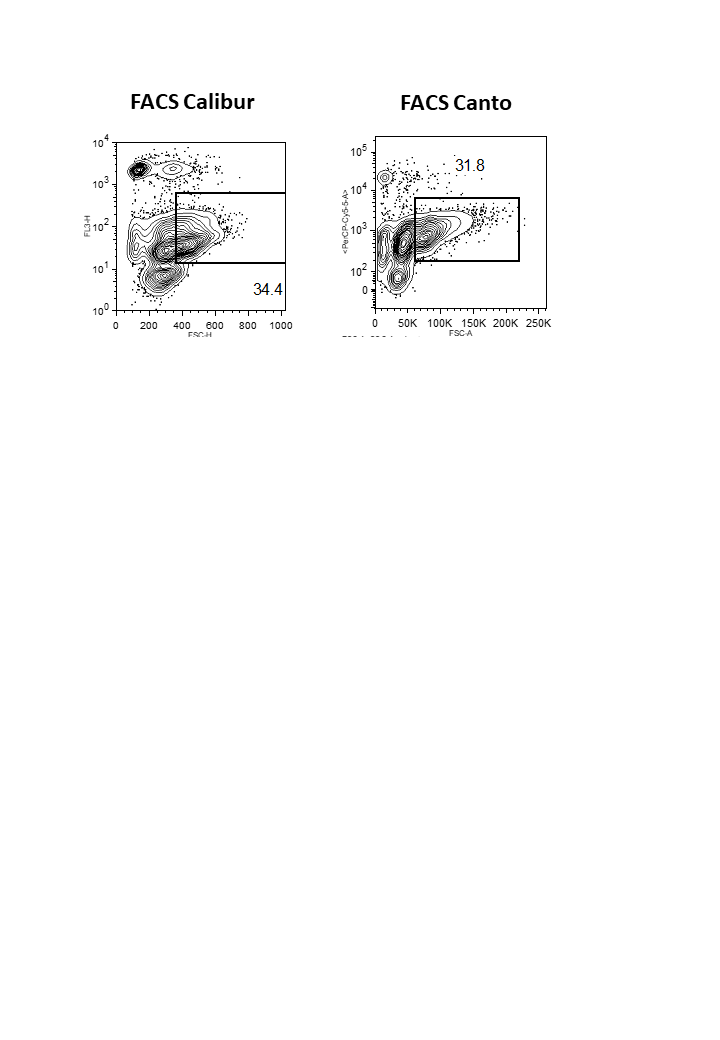

Supplement: Supplementary file 3 — Figure S3 [file ACEL-22-e13873-s002.tiff]

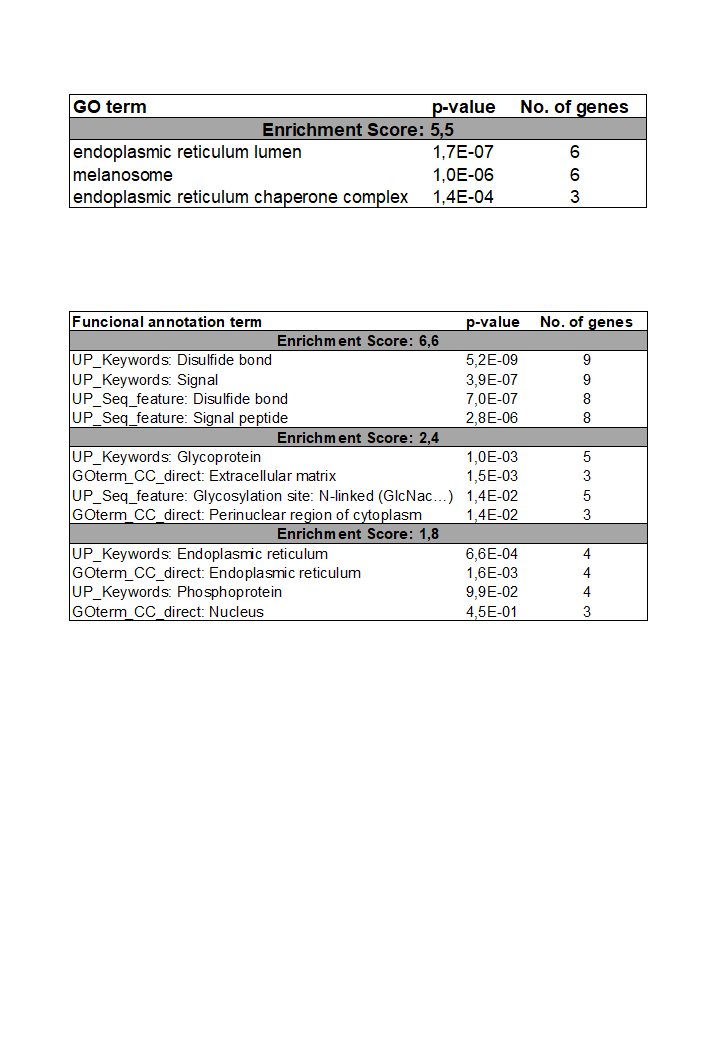

Supplement: Supplementary file 4 — Table S1 [file ACEL-22-e13873-s005.tiff]
