# Supplementary material for: Alterations of bovine nucleus pulposus cells with aging
Source: Aging Cell. 2023 May 30;22(8):e13873. doi: 10.1111/acel.13873 (PMC10410011; doi:10.1111/acel.13873)
Supplement: Supplementary file 5 — Appendix S1 [file ACEL-22-e13873-s004.docx]

**Supplementary Figure 1:** Alizarin Red staining of young and old bovine NP tissue. Left image: magnification 10x and scale bar 200 µm. Right image: magnification 20x and scale bar 100 µm.

**Supplementary Figure 2:** Gating strategy used to quantify the percentage cells expressing different surface markers in young and old bovine NP cells.

**Supplementary Figure 3:** Comparison between bovine NP cell analysis with the parameter FSC-H (in FACSCalibur) and FSC-A (in FACSCanto).

**Supplementary Table 1:** Proteins clustering according to functional similarity.

**Supplementary Table 2:** Proteins clustering according to their functional significance.
